# A Review of the Role of Food and the Food System in the Transmission and Spread of Ebolavirus

**DOI:** 10.1371/journal.pntd.0004160

**Published:** 2015-12-03

**Authors:** Erin Mann, Stephen Streng, Justin Bergeron, Amy Kircher

**Affiliations:** Food Protection and Defense Institute, University of Minnesota, Saint Paul, Minnesota, United States of America; Emory University, UNITED STATES

## Abstract

The current outbreak of Ebola virus disease (EVD) centered in West Africa is the largest in history, with nearly ten times more individuals contracting the disease than all previous outbreaks combined. The details of human-to-human and zoonotic ebolavirus transmission have justifiably received the largest share of research attention, and much information exists on these topics. However, although food processing—in the form of slaughtering and preparing wildlife for consumption (referred to as bushmeat)—has been implicated in EVD outbreaks, the full role of food in EVD spread is poorly understood and has been little studied. A literature search was undertaken to assess the current state of knowledge regarding how food can or may transmit ebolaviruses and how the food system contributes to EVD outbreak and spread. The literature reveals surprising preliminary evidence that food and the food system may be more implicated in ebolavirus transmission than expected and that further research is urgently needed.

## Introduction

The ongoing Ebola virus disease (EVD) outbreak in West Africa is the largest in history. As of September 2015, over 28,000 cases had been reported, active transmission was occurring in three countries, and seven additional countries experienced isolated cases [1]. Before 2015, less than 2,500 cases of EVD had been reported since the first known outbreak in 1976 [2]. The current outbreak has seen cases of EVD in countries that had never experienced it, including the United States, Spain, the United Kingdom, and Italy [3–6]. The world is connected in many ways—not the least of which is through food—and the world’s interconnectedness means crises in one corner of the globe can have far-reaching effects. While perhaps not apparent at first, a number of connections exist between EVD outbreaks and food. These connections can be found along the continuum of an outbreak: at its origins and as potential drivers for ongoing transmission. This article reviews what is currently known about how food can transmit ebolaviruses and how the food system contributes to EVD outbreak and spread.

## Methods

Online databases, including PubMed, Scopus, Web of Science (all databases), and Google (Scholar, News, Web) were searched for articles containing the word or word stem “ebola” and any of the following: “food,” “bushmeat,” “fomite,” “inanimate,” “environment,” “viability,” “virus survival,” “agriculture,” and “livestock.” Articles were screened for relevancy to discovering food or food system interaction with ebolavirus as well as virus survival in the environment. References in relevant articles were also screened. Additional search terms identified in the first stage screening were used to conduct subsequent searches of the above databases for “ebola” occurring with “deforestation,” “swine,” “pigs,” “mangoes,” “domestic animals,” “dogs,” and “poultry.” Topics were identified from the discovered literature and key and representative sources were included in the article.

## Animal Food Products and Ebolavirus Transmission

### Bushmeat

The exact nature of animal-to-human transmission of ebolaviruses is not often known. However, the harvesting of one food product is directly related to ebolavirus transmission. Specifically, the hunting and butchering of wildlife for food, typically referred to as “bushmeat,” exposes humans to blood and other fluids of potentially infected animals. Ebolaviruses have been detected in a number of wildlife species harvested as bushmeat, including rodents, bats, shrews, duikers, and nonhuman primates [7–9]. The Centers for Disease Control and Prevention (CDC) notes that human ebolavirus infections have been associated with handling and eating infected animals [9]. A series of EVD outbreaks during 2001–2003 in Gabon and the Republic of the Congo began after humans handled the carcasses of gorillas, chimpanzees, and duikers infected with the virus [10]. Likewise, the first human victim of an EVD outbreak in the Democratic Republic of the Congo in 2007 had purchased freshly killed bats from local hunters prior to falling ill [11].

Bushmeat harvesting and consumption is a proven high-risk activity, and it remains a risk for new cases. Studies have found that bushmeat is an important source of cash income and a food source in West Africa, particularly during times of economic hardship [12]. Bushmeat may also pose a risk to countries not currently experiencing an outbreak. Demand exists for bushmeat in the US and European Union and drives smuggling of the product. Estimates of how much bushmeat enters the US are difficult to obtain because of the market’s illicit nature. One study determined 80% of bushmeat confiscated upon entry into the US originated from West Africa [13]. Furthermore, the same study identified a 4-fold increase in bushmeat confiscation when “enhanced surveillance” was used, suggesting that routine surveillance may miss a significant amount. Studies of illegal bushmeat importation into Europe via commercial flights have begun to quantify the amount entering European countries. An estimated 273 tons of bushmeat enter France annually via Charles de Gaulle airport through personal baggage [14]. A smaller amount (8.6 tons) is estimated to enter Switzerland annually through commercial passenger flights [15].

The international market of illegal bushmeat poses public health risks [16]. A 2012 study discovered zoonotic pathogens in confiscated bushmeat, illustrating the risk of human disease transmission that smuggled bushmeat poses [17]. Furthermore, a study of macaques determined that Ebola virus (EBOV) remained viable up to seven days after death [18]. In light of the EVD outbreak and the ongoing problem of smuggled bushmeat, the European Food Safety Authority (EFSA) attempted to assess the risk of human transmission of EBOV via bushmeat. Unfortunately, EFSA concluded that it was not possible to determine the risk, given a lack of data and knowledge [19].

### Livestock

It is hypothesized that livestock could be a possible ebolavirus reservoir, given the number of human cases in previous outbreaks in which no contact with either other infected humans or diseased animal carcasses was found [20], but no cases of this occurring have been reported. Horses, sheep, and goats have been experimentally infected with EBOV [21,22]. Guinea pigs, consumed in several countries, are commonly infected in ebolavirus research. Cattle and chicken have tested positive for EBOV antibodies in early surveillance studies in the Central African Republic, but the results are considered inconclusive because of testing weaknesses [23]. There is strong evidence that dogs, which are consumed in some countries—including some with EVD outbreaks—can become infected with EBOV naturally [24]. The Food and Agriculture Organization (FAO) has characterized current knowledge by stating, “Information is extremely limited on the ability of the Ebola virus to infect livestock like cattle, sheep and goats or chickens” [25].

However, evidence does exist linking ebolaviruses and pigs: In 2008, Reston virus was discovered in domestic pigs in the Philippines [26] and in China in 2011 [27]. This virus is an ebolavirus not pathogenic to humans, but it has infected them [26,28]. Recent studies have also shown that swine can become infected with the highly pathogenic EBOV. Once the pigs became infected they developed clinical symptoms and transmitted the virus to healthy pigs [29]. Another study confirmed this susceptibility and also discovered infected pigs could transmit the virus to nonhuman primates [30]. Additional studies note that EVD outbreaks in Uganda occur in areas with high densities of swine [31] and correlate with seasonally high periods of pork consumption [20]. Furthermore, EVD outbreaks in the Democratic Republic of the Congo have been reported to immediately follow large mortality events of swine [32]. If swine are contributing to EVD etiology, either by transmitting the virus to humans or to another human-infecting animal, further outbreaks may be in store in sub-Saharan countries where swine production is growing. This is because increased production is occurring in areas adjacent to or overlapping the habitat of bats [20], who are increasingly being identified as the key ebolavirus reservoir [33].

## Plant Food Products and Ebolavirus Transmission

### Consumption of Contaminated Plant Food Products

There are no known cases of EVD involving virus transmission via consumption of a plant food product contaminated with the virus. However, in the same way that an ebolavirus may be transmitted to nonhuman primates through fruit partially eaten by bats [34], there is concern that a similar pathway may exist for human transmission. Some experts speculate that ebolavirus could be transmitted via bat saliva or feces on fruit such as mangoes or guava [35,36]. This mechanism has been experimentally verified for bat transmission of Marburg virus, a member of the Filoviridae family with ebolaviruses [37]. Notably, in the village of the two-year-old child identified as the index case of the current outbreak [38], the children played a game finding partially eaten mangoes dropped by bats [39]. Thus, it is plausible the child could have handled and even eaten a recovered mango with bat saliva on it. The concern is great enough that in the Ebola-affected nations, UNICEF advises not to eat mangoes that “have been bitten by bats” [40]. A similar mode of transmission has been documented in a different virus, Nipah virus (NiV), whose reservoir host is also the fruit bat [41]. A second EFSA risk assessment examined the risk of transmission involving raw food products other than bushmeat. Focusing primarily on fruits and vegetables, it concluded the lack of knowledge and data made it impossible to quantify the risk [42].

### Fomite Transmission Involving Plant Food Products

Ebolavirus is typically spread among humans through direct contact, but fomite transmission is also possible [43,44]. (Authors’ note: when food acts as fomite, it is often referred to as a “vehicle.” We have chosen to remain with the first term for this sense.) Fomite transmission risk is low in health care settings when decontamination procedures are in place [45]. Risks in other settings, including food harvest and production ones, are not well understood. No published literature exists specifically on the risk of plant food products serving as fomites in ebolavirus transmission. However, other viruses are known to be transmitted via food contaminated by food preparers that can subsequently infect individuals after they consume or handle the food. For example, individuals infected with hepatitis A virus can contaminate food at several points between cultivation, harvest, processing, and preparation [46]. While hepatitis A transmission involving food typically occurs via consumption, fomite transmission of the virus is also possible [47]. Another example is Lassa fever, an acute viral hemorrhagic fever closely related to EVD. Lassa fever is transmitted to humans through contact with food or objects contaminated with urine or feces from rodents infected with the virus [48]. It is therefore plausible that contaminated food (even when not consumed) could facilitate virus transmission, including the ebolaviruses, the same as other inanimate surfaces. If this is so, EVD could be spread through food handling—both between individuals working with food products and to end consumers.

Fomite transmission ultimately depends on virus survival in the environment [49]. Studies have determined that EBOV can survive outside of a host between several days to over three weeks, and virus survival is better at lower temperatures [50,51]. EBOV can also survive multiple freeze and thaw cycles [52] but is sensitive to heat and appears to be sensitive to acidic pH [53,54]. It is important to note that fomite transmission involving raw food products has not been documented. However, this area requires further research, as the consequences of any successful transmission and resultant infection would likely be severe. A summary of articles that examined ebolavirus environmental survival and sensitivities relevant to food is provided in Table 1.

**Table 1:** Articles examining Ebolavirus environmental survival and sensitivities relevant to food.

Author	Year	Key Area(s) Examined
Bray et al. [54]	1999	Thermal inactivation
Chepurnov et al. [52]	1995	Impact of freeze/thaw cycles
Mitchell et al. [53]	1984	Thermal inactivation; pH
Piercy et al. [51]	2010	Natural decay
Sagripanti et al. [50]	2010	Natural decay

## Other Ebolavirus and Food Considerations

### Public Health Consequences of Landscape Changes Caused by Food Production

Food systems may also contribute to EVD transmission indirectly. Specifically, the consequences of food production may promote environmental conditions that lead to increased human interaction with wildlife species that carry ebolaviruses. Deforestation has taken a toll on West African forests in recent years, including serious losses to the Guinean rainforest surrounding Guéckédou—the town in which the first case of the current EVD outbreak was identified [38]. This deforestation has been driven by increases in agriculture, including production of cocoa and palm oil [55]. These environmental changes driven by food production can result in serious public health consequences. For example, experts have noted that deforestation and increased human activities in tropical African forests may lead to increased contact between humans and ebolavirus reservoir species [56]. These landscape changes may have increased human activity in the forest and promoted contact with natural animal reservoirs of ebolavirus. Interestingly, these changes may have also increased animal activity, as bats are also attracted to palm oil plantations [57,58]. Experts have already begun exploring the connections between the latest EVD outbreak and ecological disruptions, including those related to agriculture [59].

### Additional Swine Concerns

An EVD outbreak among farmed swine would pose a number of concerns. First, depending on the scale of the outbreak, there could be significant animal loss with severe economic consequences. A worst-case-scenario example would be the 2010 foot and mouth disease outbreak in South Korea, during which over 3 million swine were slaughtered and buried at a cost over US$2 billion [60,61]. Second, there would likely be fear and panic among consumers regarding EVD as a foodborne illness, leading to significant market disruption. While it is unknown whether the virus can be transmitted through the meat of infected livestock, there would undoubtedly be a significant drop in consumer confidence and demand. During the 2009 Influenza A virus H1N1 outbreak, North American pork producers suffered major losses from lost sales [62] because of the initial naming of the virus as “swine flu”—even though the virus could not be contracted from eating pork [63]. There is also concern of the risk of ebolavirus becoming endemic in wild pigs, which are known to already carry numerous human disease-causing pathogens [64,65]. In feral swine, the disease would be much more difficult to manage and eradicate, and they could potentially serve as a continual reinfection source to animals and humans.

An additional concern is that swine could serve as sites for new, more harmful strains of the virus to arise [66]. Pigs’ role in facilitating the emergence of new flu strains that infect humans and other animals is well documented [67–69]. This antigenic shift in influenza viruses in pigs cannot occur with ebolaviruses, however, for antigenic shift by definition occurs in segmented viruses, and the ebolaviruses are non-segmented. RNA recombination can and does occur often among non-segmented viruses, leading to new strains. This is thought to be exceedingly rare among negative-stranded RNA viruses such as ebolaviruses [70,71], and yet it has been shown to occur, including in EBOV [72]. Furthermore, an ebolavirus could become more pathogenic via swine through other means. Its pathogenicity can be increased by intentional serial infection among laboratory animals [73,74]. Theoretically, this same increase in lethality could occur naturally through successive natural infections of swine [75].

### Harvest, Transport, and Trade

If ebolaviruses truly remain infectious on fomites for several weeks in a real-world environment, there are implications for the global food system from harvest to the end consumer. The agriculture sector is vital to the economies and labor forces of Sierra Leone, Liberia, and Guinea. According to the FAO, 58% to 78% of the total labor force participated in agriculture in 2014 [76]. Assuming the virus is even introduced into a food transport chain (domestic or international) in the first place, sufficient viability to pose a risk to those along the way and at the end destination depends on many factors. These include virus survival under varying environments and transport conditions, including temperature, humidity, sunlight exposure, product processing methods, and trip duration.

A plausible scenario for this sort of transmission could involve an enclosed, refrigerated shipping container on a cargo ship transporting food products. Surfaces of the shipping container and its contents could become contaminated through several possible routes: a symptomatic individual could handle the shipping container or its contents shortly before export, raw bushmeat could be smuggled in the shipping container, or an infected animal such as a rat or bat could contaminate surfaces through droppings. In a worst-case scenario, the virus would survive the duration of the shipment and expose those handling the container and product upon arrival.

### Food Insecurity in West Africa

The EVD outbreak in West Africa has resulted in second-order effects in food, including regional food insecurity. In September 2014, the FAO reported severe disruptions in food availability in Guinea, Liberia, and Sierra Leone because of quarantine-imposed travel restrictions on sellers (which limited supply) and consumers (which impeded access) and panic buying (which decreased supply) [77]. Also, these constraints on supply and increase in demand likely contributed to dramatic price increases, which further curtailed access. In addition, FAO predicts deteriorating food security because of reduced food production and harvests caused by farm labor shortages. Finally, income lost from reduced cash crop production has decreased many consumers’ food purchasing power. These conditions have led the United Nations to declare that West Africa is “on the brink of a major food crisis” [78]. Increasing food insecurity, however, could lead to more bushmeat harvesting and potentially to further outbreaks.

## Conclusions and Research Needs

**Fig 1:**
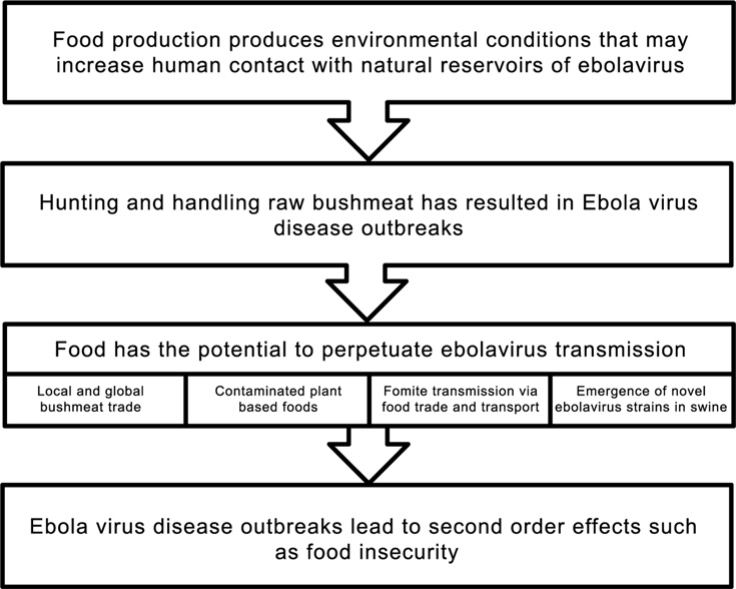
Ebolavirus, EVD outbreaks, and food.

Unlike past EVD outbreaks, many of which never made international headlines, the current outbreak continues without a clear end in sight. For example, Ebola transmission in Liberia appeared to have ended in spring 2015 only to appear several weeks later [79]. Authorities have been challenged with an evolving situation requiring public health messages to be refined. For example, the CDC initially released a “Facts about Ebola in the U.S.” infographic that stated, “You can’t get Ebola through food,” but in late 2014 updated the infographic to read instead (emphasis ours), “You can’t get Ebola through *food grown or legally purchased in the U*.*S*.” [80]. Likewise, the scientific community has responded with significant contributions to the state of knowledge about ebolaviruses and EVD, but despite important new research, much remains unknown about ebolavirus transmission [81]. Food has the potential to contribute to EVD transmission, particularly in the harvesting of bushmeat. Fig 1 outlines the ways in which food is connected to EVD outbreaks.

An examination of other roles leads to more questions than answers; however, the need for these answers is more important than ever. Research is needed in a number of areas. First, more data and research is needed to quantify the illegal bushmeat trade, identify transport routes and high-risk species involved, and perform a detailed risk assessment. Second, we need to better understand the potential role of swine in ebolavirus transmission, including for the emergence of new virus strains. Third, further information is needed on whether dogs can harbor and transmit ebolaviruses. Fourth, more studies on virus survivability in the environment are needed. Specifically, better data on survival length and environmental conditions that support survival in real-world settings is needed. This information could be applied across settings, including food harvest and transport. Fifth, research is needed on the role that plant food products could play in ebolavirus transmission. For example, we need better data on the risk of human infection via fruit contaminated by bats. Finally, if further research finds that food plays a greater role in ebolavirus transmission than previously believed, risk communication strategies need to be carefully developed.

**Key Learning Points**

- The role of harvesting of wildlife for consumption (bushmeat) in EVD outbreaks is well understood, but the full role of food in ebolavirus transmission has not been researched and is not well understood.
- Ebolavirus transmission via fomites, including food, is possible, but more research is needed to understand whether transmission via fomites or food is likely to result in perpetuating an outbreak.
- Evidence suggests that food and the food supply may be directly and indirectly implicated in all stages of an EVD outbreak—the initial outbreak, continued transmission, and ramifications of widespread disease.

**Top Five Papers**

1. Bair-Brake H, Bell T, Higgins A, Bailey N, Duda M, Shapiro S, et al. Is that a rodent in your luggage? A mixed method approach to describe bushmeat importation into the United States. Zoonoses Public Health. 2014 Mar;61(2):97–104.
2. Francesconi P, Yoti Z, Declich S, Onek PA, Fabiani M, Olango J, et al. Ebola hemorrhagic fever transmission and risk factors of contacts, Uganda. Emerg Infect Dis. 2003 Nov;9(11):1430–7.
3. Gonzalez JP, Pourrut X, Leroy E. Ebolavirus and other filoviruses. Curr Top Microbiol Immunol. 2007;315:363–87.
4. Smith KM, Anthony SJ, Switzer WM, Epstein JH, Seimon T, Jia H, et al. Zoonotic Viruses Associated with Illegally Imported Wildlife Products. PLoS ONE. 2012 Jan 10;7(1):e29505.
5. Weingartl HM, Embury-Hyatt C, Nfon C, Leung A, Smith G, Kobinger G. Transmission of Ebola virus from pigs to non-human primates. Sci Rep. 2012;2:811.

## References

[1] Centers for Disease Control and Prevention. 2014 Ebola Outbreak in West Africa—Case Counts | Ebola Hemorrhagic Fever | CDC [Internet]. 2015 [cited 23 Sep 2015]. http://www.cdc.gov/vhf/ebola/outbreaks/2014-west-africa/case-counts.html

[2] Centers for Disease Control and Prevention. Outbreaks Chronology: Ebola Virus Disease: Known Cases and Outbreaks of Ebola Virus Disease, in Reverse Chronological Order [Internet]. 20 Jan 2015 [cited 22 Jan 2015]. http://www.cdc.gov/vhf/ebola/outbreaks/history/chronology.html

[3] Centers for Disease Control and Prevention. Cases of Ebola Diagnosed in the United States [Internet]. [cited 13 Jul 2015]. http://www.cdc.gov/vhf/ebola/outbreaks/2014-west-africa/united-states-imported-case.html

[4] World Health Organization. Ebola virus disease–Spain. WHO [Internet]. 9 Oct 2014 [cited 13 Jul 2015]. http://www.who.int/csr/don/09-october-2014-ebola/en/

[5] World Health Organization. First confirmed Ebola case in United Kingdom [Internet]. 1 Feb 2015 [cited 13 Jul 2015]. http://www.euro.who.int/en/health-topics/emergencies/ebola-outbreak-2014/news/news/2014/12/first-confirmed-ebola-case-in-united-kingdom

[6] World Health Organization. First confirmed Ebola patient in Italy [Internet]. 14 May 2015 [cited 13 Jul 2015]. http://www.euro.who.int/en/health-topics/emergencies/ebola-outbreak-2014/news/news/2015/05/first-confirmed-ebola-patient-in-italy

[7] Morvan JM, Deubel V, Gounon P, Nakouné E, Barrière P, Murri S, et al Identification of Ebola virus sequences present as RNA or DNA in organs of terrestrial small mammals of the Central African Republic. Microbes Infect. 1999;1: 1193–1201. 10.1016/S1286-4579(99)00242-7 10580275

[8] Leroy EM, Rouquet P, Formenty P, Souquière S, Kilbourne A, Froment J-M, et al Multiple Ebola Virus Transmission Events and Rapid Decline of Central African Wildlife. Science. 2004;303: 387–390. 10.1126/science.1092528 14726594

[9] Centers for Disease Control and Prevention. Ebolavirus Ecology [Internet]. 1 Aug 2014. http://www.cdc.gov/vhf/ebola/resources/virus-ecology.html

[10] Rouquet P, Froment J-M, Bermejo M, Kilbourn A, Karesh W, Reed P, et al Wild animal mortality monitoring and human Ebola outbreaks, Gabon and Republic of Congo, 2001–2003. Emerg Infect Dis. 2005;11: 283–290. 10.3201/eid1102.040533 15752448 PMC3320460

[11] Leroy EM, Epelboin A, Mondonge V, Pourrut X, Gonzalez J-P, Muyembe-Tamfum J-J, et al Human Ebola Outbreak Resulting from Direct Exposure to Fruit Bats in Luebo, Democratic Republic of Congo, 2007. Vector-Borne Zoonotic Dis. 2009;9: 723–728. 10.1089/vbz.2008.0167 19323614

[12] Björn Schulte-Herbrüggen GC. The importance of bushmeat in the livelihoods of west african cash-crop farmers living in a faunally-depleted landscape. PLoS ONE. 2013;8: e72807 10.1371/journal.pone.0072807 23977355 PMC3745405

[13] Bair-Brake H, Bell T, Higgins A, Bailey N, Duda M, Shapiro S, et al Is that a rodent in your luggage? A mixed method approach to describe bushmeat importation into the United States. Zoonoses Public Health. 2014;61: 97–104. 10.1111/zph.12050 23678947

[14] Chaber A-L, Allebone-Webb S, Lignereux Y, Cunningham AA, Marcus Rowcliffe J. The scale of illegal meat importation from Africa to Europe via Paris. Conserv Lett. 2010;3: 317–321. 10.1111/j.1755-263X.2010.00121.x

[15] Falk H, Dürr S, Hauser R, Wood K, Tenger B, Lörtscher M, et al Illegal import of bushmeat and other meat products into Switzerland on commercial passenger flights. Rev Sci Tech Int Off Epizoot. 2013;32: 727–739. 10.20506/rst.32.2.2221 24761726

[16] Gómez A, Aguirre AA. Infectious Diseases and the Illegal Wildlife Trade. Ann N Y Acad Sci. 2008;1149: 16–19. 10.1196/annals.1428.046 19120165

[17] Smith KM, Anthony SJ, Switzer WM, Epstein JH, Seimon T, Jia H, et al Zoonotic Viruses Associated with Illegally Imported Wildlife Products. PLoS ONE. 2012;7: e29505 10.1371/journal.pone.0029505 22253731 PMC3254615

[18] Prescott J, Bushmaker T, Fischer R, Miazgowicz K, Judson S, Munster VJ. Postmortem Stability of Ebola Virus. Emerg Infect Dis. 2015;21 10.3201/eid2105.150041 25897646 PMC4412251

[19] European Food Safety Authority. An update on the risk of transmission of Ebola virus (EBOV) via the food chain. 2014.

[20] Atherstone C, Roesel K, Grace D. Ebola risk assessment in the pig value chain in Uganda [Internet]. ILRI; 2014. https://cgspace.cgiar.org/handle/10568/41667

[21] Krasnianskiĭ B, Mikhaĭlov V, Borisevich I, Gradoboev V, Evseev A, Va P. Preparation of hyperimmune horse serum against Ebola virus. Vopr Virusol. 1994;40: 138–140. http://europepmc.org/abstract/med/7676681 7676681

[22] Kudoyarova-Zubavichene NM, Sergeyev NN, Chepurnov AA, Netesov SV. Preparation and Use of Hyperimmune Serum for Prophylaxis and Therapy of Ebola Virus Infections. J Infect Dis. 1999;179: S218–S223. 10.1086/514294 9988187

[23] Gonzalez J-P, Herbreteau V, Morvan J, Leroy E. Ebola virus circulation in Africa: a balance between clinical expression and epidemiological silence. Bull Société Pathol Exot. 2005;98: 210–217. http://hal.ird.fr/hal-00376214/ 16267963

[24] Allela L, Bourry O, Pouillot R, Délicat A, Yaba P, Kumulungui B, et al Ebola Virus Antibody Prevalence in Dogs and Human Risk. Emerg Infect Dis. 2005;11: 385–390. 10.3201/eid1103.040981 15757552 PMC3298261

[25] Food and Agriculture Organization. Frequently asked questions on Ebola virus disease. Food and Agriculture Organization of the United Nations [Internet]. 10 Feb 2014 [cited 18 Nov 2014]. http://www.fao.org/emergencies/fao-in-action/stories/stories-detail/en/c/251862/

[26] Barrette RW, Metwally SA, Rowland JM, Xu L, Zaki SR, Nichol ST, et al Discovery of Swine as a Host for the Reston ebolavirus. Science. 2009;325: 204–206. 10.1126/science.1172705 19590002

[27] Pan Y, Zhang W, Cui L, Hua X, Wang M, Zeng Q. Reston virus in domestic pigs in China. Arch Virol. 2014;159: 1129–1132. 10.1007/s00705-012-1477-6 22996641

[28] Centers for Disease Control and Prevention. Update: Filovirus Infection Associated with Contact with Nonhuman Primates or Their Tissues. MMWR. 1990;69: 404–405. http://www.cdc.gov/mmwr/preview/mmwrhtml/00001646.htm 2112686

[29] Kobinger GP, Leung A, Neufeld J, Richardson JS, Falzarano D, Smith G, et al Replication, Pathogenicity, Shedding, and Transmission of Zaire ebolavirus in Pigs. J Infect Dis. 2011; 204(2):200–8. 10.1093/infdis/jir077 21571728

[30] Weingartl HM, Embury-Hyatt C, Nfon C, Leung A, Smith G, Kobinger G. Transmission of Ebola virus from pigs to non-human primates. Sci Rep. 2012;2: 811 10.1038/srep00811 23155478 PMC3498927

[31] Weingartl HM, Nfon C, Kobinger G. Review of Ebola virus infections in domestic animals. Dev Biol Basel. 2013;135: 211–218. 10.1159/000178495 23689899

[32] Katayi K. Ebola sleuths scour DR Congo jungle for source of outbreak (L’Agence France-Press). Yahoo News. 23 Oct 2014. http://news.yahoo.com/ebola-sleuths-scour-dr-congo-jungle-source-outbreak-060715032.html. Accessed 3 Aug 2015.

[33] Olival KJ, Hayman DTS. Filoviruses in Bats: Current Knowledge and Future Directions. Viruses. 2014;6: 1759–1788. 10.3390/v6041759 24747773 PMC4014719

[34] Gonzalez JP, Pourrut X, Leroy E. Ebolavirus and other filoviruses. Curr Top Microbiol Immunol. 2007;315: 363–387. 17848072 10.1007/978-3-540-70962-6_15 PMC7121322

[35] Fox M. Ebola Outbreak “Tip of the Iceberg,” Experts Say. NBC News [Internet]. 22 Jun 2014 [cited 2 Dec 2014]. http://www.nbcnews.com/storyline/ebola-virus-outbreak/ebola-outbreak-tip-iceberg-experts-say-n137081

[36] Alexander KA, Sanderson CE, Marathe M, Lewis BL, Rivers CM, Shaman J, et al What factors might have led to the emergence of Ebola in West Africa? PLOS Medical Journals’ Community Blog [Internet]. 2014 [cited 2 Dec 2014]. http://blogs.plos.org/speakingofmedicine/2014/11/11/factors-might-led-emergence-ebola-west-africa/ 10.1371/journal.pntd.0003652 PMC4456362 26042592

[37] Amman BR, Jones ME, Sealy TK, Uebelhoer LS, Schuh AJ, Bird BH, et al Oral shedding of Marburg virus in experimentally infected Egyptian fruit bats (Rousettus Aegyptiacus). J Wildl Dis. 2015;51(1):113–24. http://www.jwildlifedis.org/doi/abs/10.7589/2014-08-198 10.7589/2014-08-198 25375951 PMC5022530

[38] Baize S, Pannetier D, Oestereich L, Rieger T, Koivogui L, Magassouba N, et al Emergence of Zaire Ebola Virus Disease in Guinea. N Engl J Med. 2014;371: 1418–1425. 10.1056/NEJMoa1404505 24738640

[39] Coen A, Henk M. How the virus came into this world. Die Zeit. Online English Translation. Hamburg; 28 Nov 2014. http://www.zeit.de/feature/ebola-afrika-virus. Accessed 21 Jan 2015.

[40] UNICEF. Frequently Asked Questions on Ebola: For TV/radio/community/school discussions and Toll Free staffs [Internet]. UNICEF; no date. http://www.unicef.org/cbsc/files/Ebola_FAQ-SierraLeone-EN.pdf

[41] Luby SP, Gurley ES, Hossain MJ. Transmission of human infection with Nipah virus. Clin Infect Dis Off Publ Infect Dis Soc Am. 2009;49: 1743–1748. 10.1086/647951 PMC2784122 19886791

[42] European Food Safety Authority. An update on the risk of transmission of Ebola virus (EBOV) via the food chain–Part 2. EFSA J. 2015;13: 4042–4058. 10.2903/j.efsa.2015.4042

[43] Francesconi P, Yoti Z, Declich S, Onek PA, Fabiani M, Olango J, et al Ebola hemorrhagic fever transmission and risk factors of contacts, Uganda. Emerg Infect Dis. 2003;9: 1430–1437. 10.3201/eid0911.030339 14718087 PMC3035551

[44] Roels TH, Bloom AS, Buffington J, Muhungu GL, Mac Kenzie WR, Khan AS, et al Ebola hemorrhagic fever, Kikwit, Democratic Republic of the Congo, 1995: risk factors for patients without a reported exposure. J Infect Dis. 1999;179 Suppl 1: S92–97. 10.1086/514286 9988170

[45] Bausch DG, Towner JS, Dowell SF, Kaducu F, Lukwiya M, Sanchez A, et al Assessment of the risk of Ebola virus transmission from bodily fluids and fomites. J Infect Dis. 2007;196 Suppl 2: S142–147. 10.1086/520545 17940942

[46] Acheson D, Fiore AE. Hepatitis A Transmitted by Food. Clin Infect Dis. 2004;38: 705–715. 10.1086/381671 14986256

[47] Boone SA, Gerba CP. Significance of Fomites in the Spread of Respiratory and Enteric Viral Disease. Appl Environ Microbiol. 2007;73: 1687–1696. 10.1128/AEM.02051-06 17220247 PMC1828811

[48] WHO | Lassa fever. WHO [Internet]. 2015 [cited 23 Jul 2015]. http://www.who.int/mediacentre/factsheets/fs179/en/

[49] Sinclair R, Boone SA, Greenberg D, Keim P, Gerba CP. Persistence of Category A Select Agents in the Environment. Appl Environ Microbiol. 2008;74: 555–563. 10.1128/AEM.02167-07 18065629 PMC2227740

[50] Sagripanti J-L, Rom AM, Holland LE. Persistence in darkness of virulent alphaviruses, Ebola virus, and Lassa virus deposited on solid surfaces. Arch Virol. 2010;155: 2035–2039. 10.1007/s00705-010-0791-0 20842393

[51] Piercy TJ, Smither SJ, Steward JA, Eastaugh L, Lever MS. The survival of filoviruses in liquids, on solid substrates and in a dynamic aerosol. J Appl Microbiol. 2010;109: 1531–1539. 10.1111/j.1365-2672.2010.04778.x 20553340

[52] Chepurnov AA, Chuev IP, P’iankov OV, Efimova IV. [The effect of some physical and chemical factors on inactivation of the Ebola virus]. Vopr Virusol. 1995;40: 74–76. 7762236

[53] Mitchell SW, McCormick JB. Physicochemical inactivation of Lassa, Ebola, and Marburg viruses and effect on clinical laboratory analyses. J Clin Microbiol. 1984;20: 486–489. 6490832 10.1128/jcm.20.3.486-489.1984 PMC271356

[54] Bray M, Davis K, Geisbert T, Schmaljohn C, Huggins J. A mouse model for evaluation of prophylaxis and therapy of Ebola hemorrhagic fever. J Infect Dis. 1999;179 Suppl 1: S248–258. 10.1086/514292 9988191

[55] Gockowski J, Sonwa D. Cocoa Intensification Scenarios and Their Predicted Impact on CO2 Emissions, Biodiversity Conservation, and Rural Livelihoods in the Guinea Rain Forest of West Africa. Environ Manage. 2011;48: 307–321. 10.1007/s00267-010-9602-3 21191791

[56] Muyembe-Tamfum JJ, Mulangu S, Masumu J, Kayembe JM, Kemp A, Paweska JT. Ebola virus outbreaks in Africa: past and present. Onderstepoort J Vet Res. 2012;79: 451 10.4102/ojvr.v79i2.451 23327370

[57] Wallace RG, Gilbert M, Wallace R, Pittiglio C, Mattioli R, Kock R. Did Ebola emerge in West Africa by a policy-driven phase change in agroecology? Environ Plan A. 2014;46: 2533–2542. 10.1068/a4712com

[58] Shafie NJ, Sah SAM, Latip NSA, Azman NM, Khairuddin NL. Diversity Pattern of Bats at Two Contrasting Habitat Types along Kerian River, Perak, Malaysia. Trop Life Sci Res. 2011;22: 13–22. http://www.ncbi.nlm.nih.gov/pmc/articles/PMC3819087/ 24575214 PMC3819087

[59] Bausch DG, Schwarz L . Outbreak of Ebola Virus Disease in Guinea: Where Ecology Meets Economy. PLoS Negl Trop Dis. 2014;8: e3056 10.1371/journal.pntd.0003056 25079231 PMC4117598

[60] Park J-H, Lee K-N, Ko Y-J, Kim S-M, Lee H-S, Shin Y-K, et al Control of Foot-and-Mouth Disease during 2010–2011 Epidemic, South Korea. Emerg Infect Dis. 2013;19: 655–659. 10.3201/eid1904.121320 23632094 PMC3647416

[61] Food and Agriculture Organization. FAO—Animal Production and Health. AGA News [Internet]. 25 Sep 2012 [cited 20 Jul 2015]. http://www.fao.org/ag/againfo/home/en/news_archive/2011_economic-impact-FMD.html

[62] Gietz R. Estimating the Cost of H1N1. Advances in Pork Production. Banff, Alberta; 2010. pp. 17–25. https://www.banffpork.ca/documents/039-Gietz.pdf

[63] Vincent AL, Lager KM, Harland M, Lorusso A, Zanella E, Ciacci-Zanella JR, et al Absence of 2009 Pandemic H1N1 Influenza A Virus in Fresh Pork. PLoS ONE. 2009;4: e8367 10.1371/journal.pone.0008367 20020048 PMC2791228

[64] Hutton T, DeLiberto T, Owen S, Morrison B. Disease risks associated with increasing feral swine numbers and distribution in the United States. Midwest Association of Fish and Wildlife Agencies Wildlife and Fish Health Committee; 2006 Jul.

[65] Bevins SN, Pedersen K, Lutman MW, Gidlewski T, Deliberto TJ. Consequences Associated with the Recent Range Expansion of Nonnative Feral Swine. BioScience. 2014;64: 291–299. 10.1093/biosci/biu015

[66] MacNeil A, Rollin PE. Ebola and Marburg Hemorrhagic Fevers: Neglected Tropical Diseases? PLoS Negl Trop Dis. 2012;6: e1546 10.1371/journal.pntd.0001546 22761967 PMC3385614

[67] Ma W, Lager KM, Vincent AL, Janke BH, Gramer MR, Richt JA. The Role of Swine in the Generation of Novel Influenza Viruses. Zoonoses Public Health. 2009;56: 326–337. 10.1111/j.1863-2378.2008.01217.x 19486316

[68] Torremorell M, Allerson M, Corzo C, Diaz A, Gramer M. Transmission of Influenza A Virus in Pigs. Transbound Emerg Dis. 2012;59: 68–84. 10.1111/j.1865-1682.2011.01300.x 22226050

[69] Lorusso A, Vincent AL, Gramer MR, Lager KM, Ciacci-Zanella JR. Contemporary Epidemiology of North American Lineage Triple Reassortant Influenza A Viruses in Pigs Richt JA, Webby RJ, editors. Swine Influenza. Springer Berlin Heidelberg; 2013 pp. 113–131. http://link.springer.com/chapter/10.1007/82_2011_196 10.1007/82_2011_196 PMC7120137 22266673

[70] Chare ER, Gould EA, Holmes EC. Phylogenetic analysis reveals a low rate of homologous recombination in negative-sense RNA viruses. J Gen Virol. 2003;84: 2691–2703. 10.1099/vir.0.19277–0 13679603

[71] Simon-Loriere E, Holmes EC. Why do RNA viruses recombine? Nat Rev Microbiol. 2011;9: 617–626. 10.1038/nrmicro2614 21725337 PMC3324781

[72] Wittmann TJ, Biek R, Hassanin A, Rouquet P, Reed P, Yaba P, et al Isolates of Zaire ebolavirus from wild apes reveal genetic lineage and recombinants. Proc Natl Acad Sci. 2007;104: 17123–17127. 10.1073/pnas.0704076104 17942693 PMC2040453

[73] Pereboeva L, Tkachev V, Kolesnikova L, Krendeleva L, Ryabchikova E, Smolina M. Ultrastructural-Changes of Guinea-Pig Organs in Sequential Passages of Ebola Virus. Vopr Virusol. 1993;38: 179–182. 8236945

[74] Ryabchikova E, Kolesnikova L, Smolina M, Tkachev V, Pereboeva L, Baranova S, et al Ebola virus infection in guinea pigs: presumable role of granulomatous inflammation in pathogenesis. Arch Virol. 1996;141: 909–921. 10.1007/BF01718165 8678836

[75] Bausch DG. Ebola Virus as a Foodborne Pathogen? Cause for Consideration, but Not Panic. J Infect Dis. 2011;204: 179–181. 10.1093/infdis/jir201 21571727

[76] Food and Agriculture Organization. Country Profiles [Internet]. 2015. faostat3.fao.org/

[77] FAO Global Information and Early Warning System. Grave food security concerns following the Ebola outbreak in Liberia, Sierra Leone and Guinea [Internet]. Rome: Food and Agriculture Organization of the United Nations; 2014 Sep. Report No.: 333. http://www.fao.org/3/a-i4003e.pdf

[78] United Nations. West Africa on the brink of a major food crisis as Ebola threatens food security, warns UN expert [Internet]. 2014. http://www.ohchr.org/EN/NewsEvents/Pages/DisplayNews.aspx?NewsID=15276&LangID=E

[79] World Health Organization. Ebola Situation Report—8 July 2015 [Internet]. 2015. http://apps.who.int/ebola/current-situation/ebola-situation-report-8-july-2015

[80] Centers for Disease Control and Prevention. Facts about Ebola in the U.S. [Internet]. 2015. http://www.cdc.gov/vhf/ebola/pdf/infographic.pdf

[81] Osterholm MT, Moore KA, Kelley NS, Brosseau LM, Wong G, Murphy FA, et al Transmission of Ebola Viruses: What We Know and What We Do Not Know. mBio. 2015;6: e00137–15. 10.1128/mBio.00137-15 25698835 PMC4358015

